# Impact of storage time prior to cryopreservation on mechanical properties of aortic homografts

**DOI:** 10.1007/s10561-023-10079-z

**Published:** 2023-02-27

**Authors:** Ida Axelsson, Anna Gustafsson, Hanna Isaksson, Johan Nilsson, Torsten Malm

**Affiliations:** 1 Tissue Bank Lund, Baravägen 37, 22242 Lund, Sweden; 2 Department of Cardiothoracic Surgery, Skane University Hospital, Lund, Sweden; 3 Department of Clinical Science, Cardiothoracic Surgery, Lund University, Lund, Sweden; 4 Department of Biomedical Engineering, Lund University, Lund, Sweden; 5 Department of Translational Medicine, Artificial Intelligence and Bioinformatics in Cardiothoracic Sciences, Lund University, Lund, Sweden; 6 Pediatric Cardiac Surgery Unit, Children’s Hospital, Skane University Hospital, Lund, Sweden

**Keywords:** Elastic modulus, Yield stress, Congenital cardiac surgery, Homografts

## Abstract

Optimal time spans in homograft procurement are still debatable among tissue banks and needs to be further investigated. Cell viability decreases at longer preparation intervals, but the effect on collagen and elastic fibers has not been investigated to the same extent. These fibers are of importance to the homograft elasticity and strength. The objective of this study was to analyze the mechanical properties of homograft tissue at different time spans in the procurement process. Ten aortic homografts were collected at the Tissue Bank in Lund. Twelve samples were obtained from each homograft, cryopreserved in groups of three after 2–4 days, 7–9 days, 28–30 days, and 60–62 days in antibiotic decontamination. Mechanical testing was performed with uniaxial tensile tests, calculating elastic modulus, yield stress and energy at yield stress. Two randomly selected samples were assessed with light microscopy. Procurement generated a total of 120 samples, with 30 samples in each time group. Elastic modulus and yield stress was significantly higher in samples cryopreserved after 2–4 days (2.7 MPa (2.5-5.0) and 0.78 MPa (0.68-1.0)) compared to 7–9 days (2.2 MPa (2.0-2.6) and 0.53 MPa (0.46–0.69)), *p* = 0.008 and 0.011 respectively. Light microscopy did not show any difference in collagen and elastin at different time spans. There was a significant decrease in elastic modulus and yield stress after 7 days of decontamination at 4 °C compared to 2–4 days. This could indicate some deterioration of elastin and collagen at longer decontamination intervals. Clinical significance of these findings remains to be clarified.

## Introduction

Cardiovascular homografts have been used as conduits in reconstruction of the right ventricular outflow tract since the method was first introduced by Ross et al. in 1966 (Ross and Somerville 1966). Procurement, preparation, and storage of homografts are conducted at tissue banks, with strict protocols that regulate every detail of the process. Tissue banks in Europe receive recommended guidelines from the European Directorate for the Quality of Medicine and Health Care (EDQM), but local adaptions are common, according to different experiences and traditions (Keitel 2019; Zahra et al. 2019).

According to different time spans in the procurement process, EDQM recommends a maximum of 24 h of ischemic time (time from donor death until heart procurement), a maximum of 24 h from heart procurement to preparation of homografts, and a minimum of 24 h of antibiotic decontamination prior to cryopreservation. They recommend that the total time from circulatory arrest in the donor until cryopreservation should not exceed 72 h (Keitel 2019). These guidelines appear to be based mainly on experience and tradition. There are no references to scientific evaluation of preparation times in this chapter of the EDQM recommendations. The Tissue Bank in Lund, Sweden has used longer time spans. The Tissue Bank was established in 1989 and already had many years of experience when the first European guidelines were presented in 2013. It was decided to keep guidelines already in use, accepting a maximum of 48 h of ischemic time and a total maximum of 72 h from donor death to heart preparation, allowing 24 h for transportation of the heart to the tissue bank. In alliance with the EDQM guidelines, a blood sample is required from the donor for serological testing within 24 h of death to avoid hemolysis. After preparation, antibiotic decontamination is conducted for a minimum of 24 h and a maximum of 96 h. The allowed extended time spans lead to a larger pool of possible donors, and more time to retrieve and transport the hearts to the tissue bank (Axelsson and Malm 2018; Bester et al. 2018).

Studies evaluating different time spans during procurement in relation to long-term performance of the homograft after implantation, have not been able to show any clinical disadvantages of extended procurement times (Meyns et al. 2005; Kalfa et al. 2011; Smit et al. 2015). Repeated studies have shown that prolonged time spans during procurement decrease cell viability of the homograft, but these findings have not shown any correlation to long-term outcome after implantation (Mochtar et al. 1984; Angell et al. 1989; Niwaya et al. 1995; Gall et al. 1998). The importance of other components of the homografts, such as collagen and elastin, has been given less attention. Both fibers are important for the mechanical properties of the homograft, providing strength and elasticity to the tissue (Stradins et al. 2004; Burkert et al. 2021). These parameters can be analyzed by calculating the elastic modulus and maximum strength of the tissue (Stradins et al. 2004; Lin et al. 2016; Kubíková et al. 2017; Bester et al. 2018).

The aim of this study was to evaluate homograft tissue during different time intervals in the procurement process. The objective was to determine if prolonged time during antibiotic decontamination had any effect on the mechanical properties of the homograft.

## Materials and methods

### Ethical statement

The Regional Ethical Review Board in Lund, Sweden, approved the study (Dnr 2017/133 and Dnr 2018/568).

### Homografts

Homografts were collected from the Tissue Bank in Lund. Only homografts not suitable for transplantation, due to structural impairments such as fenestration of the cusps or localized atherosclerosis, were used in the study. Homografts were collected from donors that had accepted donation for scientific purposes.

**Fig. 1:**
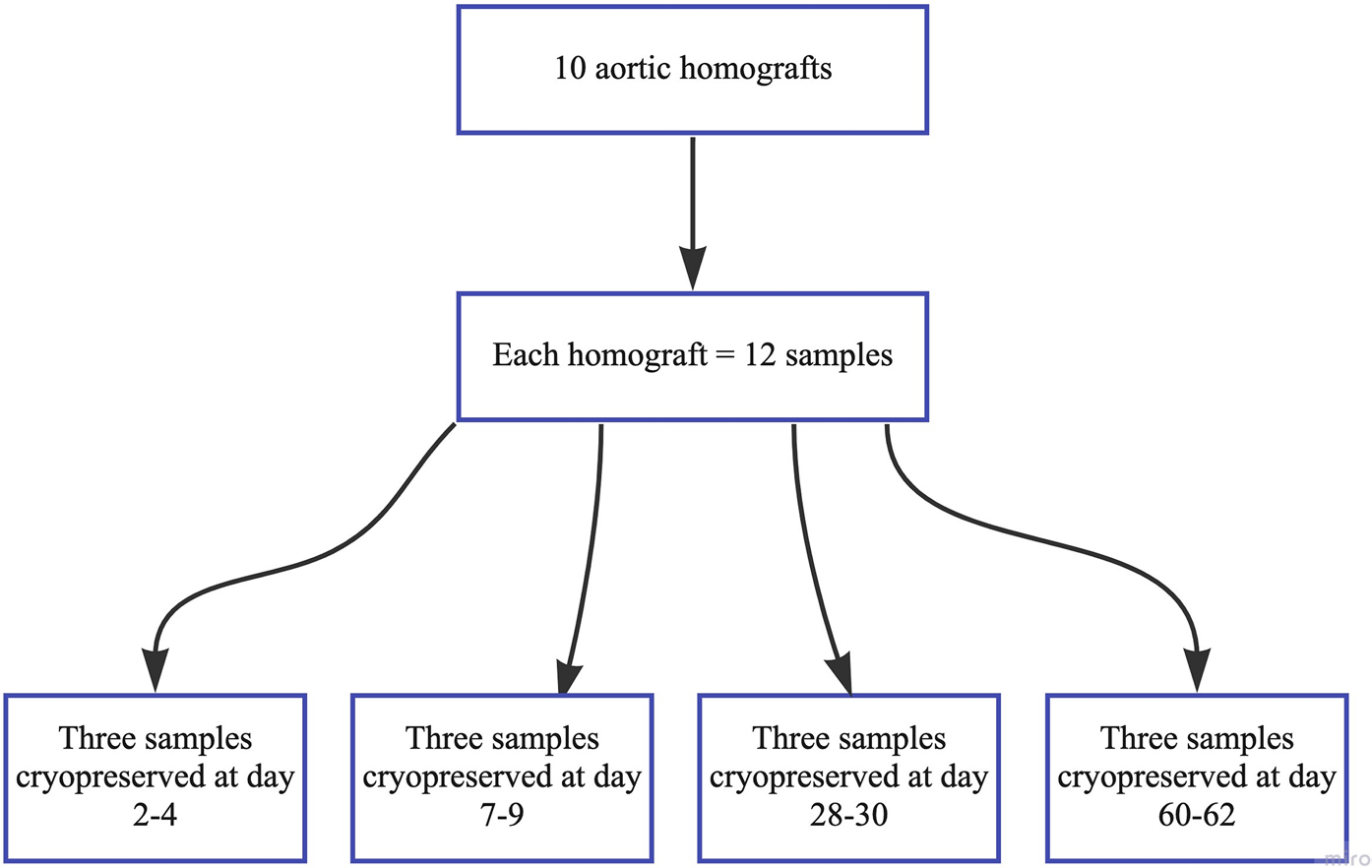
Flow chart of homograft sample collection

Ten aortic homografts were collected in 2020 and 2021 according to regular guidelines used at the Tissue Bank in Lund. Mean donor age was 43 years (SD 32–53, range 20–69). There were three multi organ donors (30%) and seven non-heart beating donors (NHBD) (70%). The mean ischemic time for NHBD was 30 h (SD 20–40, range 11–42). At preparation, the aortic vessel wall was measured with a ruler and then cut longitudinally with a surgical scissor into a total of 12 samples from each donor homograft (Fig. 1). Samples were measured to 5 mm in width and a minimum of 30 mm in height. All samples were decontaminated in an antibiotic solution at 4 °C according to standard protocols. Three of the samples were cryopreserved after 2–4 days of antibiotic decontamination (four homografts had two days, one homograft had three days and five homografts had four days of decontamination time). These samples were used as reference samples, since they were prepared, decontaminated, and cryopreserved according to the local standard protocol for homograft procurement. The remaining nine samples were left in the antibiotic solution for an extended period and cryopreserved in groups of three samples after 7–9 days, 28–30 days and 60–62 days. The group of 7–9 days was chosen to see if a small difference in time compared to the reference group would affect the result. The group of 60–62 days was chosen since this was the maximum time used when homografts were stored fresh prior to the introduction of cryopreservation in 1987 (O’Brien et al. 1987). The group of 28–30 days was chosen since it was in between the first and last group. All homografts were collected, prepared, and cryopreserved according to standard protocols, so that the homografts in the study would represent the homografts used in clinical practice. Details of these protocols are described elsewhere (Axelsson et al. 2021). Conclusively, each homograft generated 12 samples, where three samples were cryopreserved at each time point investigated. Each time point had a total of 30 samples, three replicate from each of the 10 donors (Fig. 1).

### Mechanical testing

The tensile mechanical properties of the samples were tested using a uniaxial testing machine (Instron 8511 load frame, High Wycombe, UK/MTS Test Star II controller, Minneapolis, US). Time, force, and deformation were recorded, and elastic modulus, yield stress and energy at yield stress were calculated. The elastic modulus represents the elasticity of the tissue, where higher values indicate an increased stiffness of the tissue. Yield stress represents the maximum stress (force/cross-sectional area) that can be applied to the tissue before it deforms permanently, thus when the tissue cannot return to its original state. The energy at yield stress describes the total absorbed energy at the point of yield stress.

**Fig. 2:**
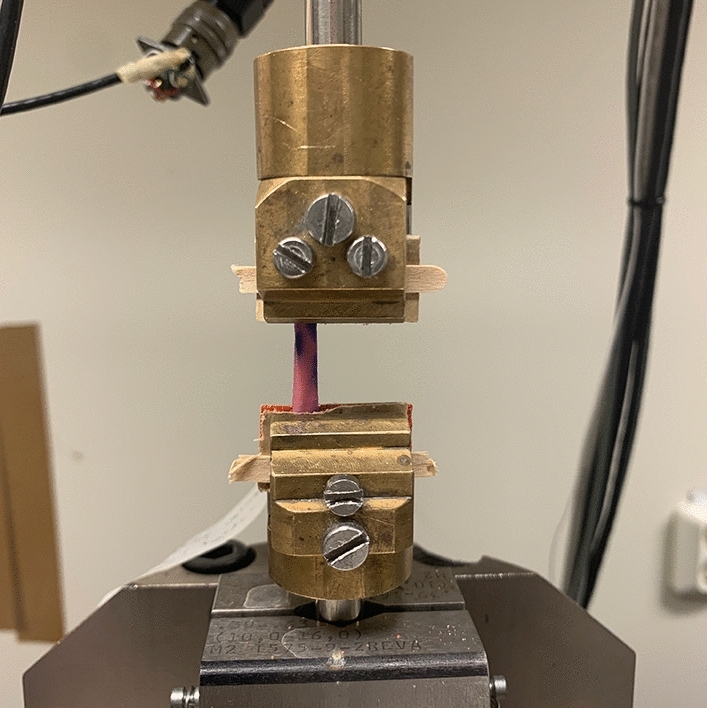
Picture of mechanical testing set-up

Tensile tests were performed at room temperature and samples were thawed from cryopreservation just before testing. Routines from thawing in the operating room were imitated, by quick thawing in water with a temperature of 37 °C, before unpacking the samples from their storing bags. Samples were mounted longitudinally in the machine with at least 10 mm distance between the grips (Fig. 2). The sample was stretched manually until no slacking was observed. Thickness and length between grips of the sample were measured with a digital caliper (Table 1). From each homograft, three replicate samples per time point were tested.

**Table 1:** Properties of samples at different time points. Three samples per homograft in each time group

	Day 2–4 (n = 27)	Day 7–9 (n = 29)		Day 28–30 (n = 29)		Day 60–62 (n = 28)	
	Median (IQR)	Median (IQR)	*p*-value^a^	Median (IQR)	*p*-value^a^	Median (IQR)	*p*-value^a^
Length (mm)	22 (17–28)	18 (17–21)	0.262	18 (14–25)	0.0125	16 (14–20)	0.0367
Width (mm)	5.1 (4.7–5.5)	4.8 (4.6–5.2)	0.358	4.9 (4.6–5.2)	0.203	4.7 (4.5–5.1)	0.0367
Thickness (mm)	1.7 (1.6–2)	1.6 (1.5–1.8)	0.610	1.6 (1.4–1.8)	0.0367	1.6 (1.4–1.9)	0.0412

^a^Differences were analyzed using Wilcoxon Signed Rank test. Groups are compared separately to the group “Day 2–4”

**Fig. 3:**
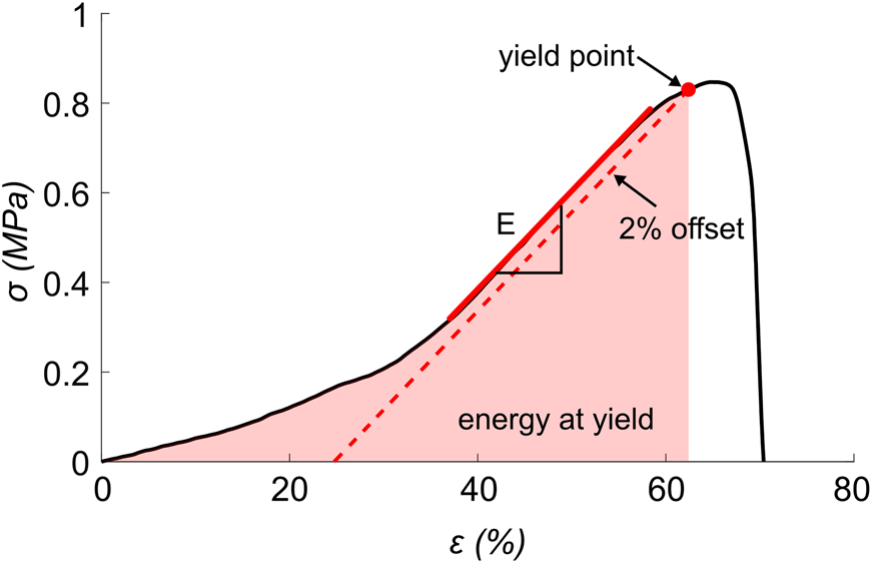
A typical stress-strain curve for a homograft sample, indicating the calculated mechanical properties Elastic modulus (E), yield stress and energy at yield. Y-axis presents stress in megapascal (σ), and x-axis presents strain (ɛ)

The specimens were tested until failure at a speed of 1 mm/s and force and displacement data were recorded. The stress was calculated by normalizing the forces with the cross-sectional area. The initial gauge length was determined as the length between the grips at 0.05 MPa stress. The strain was then calculated as the displacement divided by this gauge length. The elastic modulus was calculated as the slope of the linear part of the stress-strain curve, the yield stress was determined using a 2% offset criterion and the energy at yield was calculated as the area under the stress-strain curve until the yield point. Mechanical parameters were defined according to Fig. 3.

### Morphology

Homograft samples were assessed with light microscopy to ensure tissue quality and reveal major morphological changes at different time points. Two homografts were chosen at random and biopsies were received after mechanical testing from all time points on those homografts. Biopsies were 0,5 × 0,5 cm in size, taken from the end of the sample strip that had ruptured during mechanical testing. The ruptured area was included in the biopsies. Samples were used as a random control of the total group to reveal possible major alterations of tissue at prolonged decontamination times. More detailed morphological analysis was not the major focus of this study. Samples were fixed in formalin at 4 °C directly after collection. Samples were dehydrated with increasing concentrations of ethanol and xylene and then embedded in paraffin under vacuum. Sections of 3,5 mm were placed on slides in heated distilled water and then dried overnight in a heating cabinet. Sections were stained with hematoxylin-eosin saffron for inspection of cell nuclei, elastica van Gieson for elastin and azan for collagen. Samples were scanned with NanoZoomer-SQ Digital slide scanner (Hamamatsu, Kista, Sweden) and assessed at a computer screen through NDP.view2 Image viewing software (Hamamatsu, Kista, Sweden). Analysis was conducted with 20x magnification. Samples were compared with a control sample where biopsies had been collected from a homograft after 2, 7, 28 and 60 days in antibiotic contamination without prior cryopreservation or mechanical testing. Time points for the morphological control samples were chosen since they were available from another study conducted by the researchers. Day 2 was chosen at random since both day 1 and day 3 is available as well. However, preliminary data have not shown any major differences in morphological appearance between day 1, 2 and 3 (data not published).

**Fig. 4:**
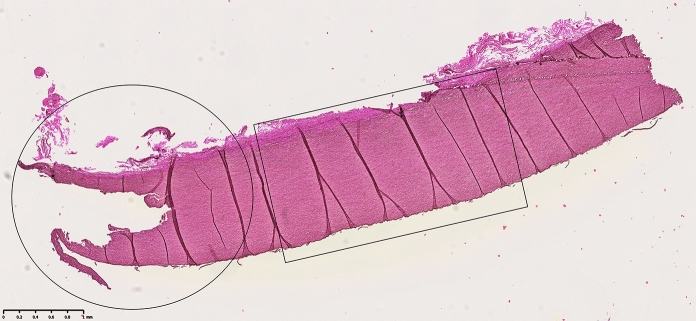
The ruptured area was clearly identified (circle). Morphological assessment was performed next to the ruptured area (rectangle)

When investigating the biopsies taken from the mechanically tested homografts, it clearly showed the point of rupture, with disrupted elastin fibers. When comparing the biopsies to the control samples, assessment was made next to the ruptured area (Fig. 4).

### Statistics

Donor characteristics are presented as means with standard deviations. Results from mechanical testing are presented as medians with an interquartile range. At each time point (2–4 days, 7–9 days, 28–30 days, and 60–62 days) there were three replicate samples per homograft. The mean value of the three samples was used for statistical analysis.

Differences in mechanical properties and sample size measurement (length, width, and thickness) were analyzed with the Wilcoxon signed rank test. A non-parametric test was selected as the data was not normally distributed. Samples from day 7–9, 28–30, and 60–62 were compared to the reference samples from day 2–4. Post-hoc test of multiple comparisons test was not conducted. Analyses were performed with Stata Statistical Software (StataCorp. 2017. Release 15. College Station, TX: StataCorp LLC, USA).

Main outcome was the differences in mechanical properties at different groups, e.g., Elastic modulus, yield stress and energy and yield stress.

## Results

### Mechanical testing

Out of the 120 samples tested, fourteen (12%) samples had missing data due to problems during testing and data collection. The remaining 106 samples were used for analysis.

**Fig. 5:**
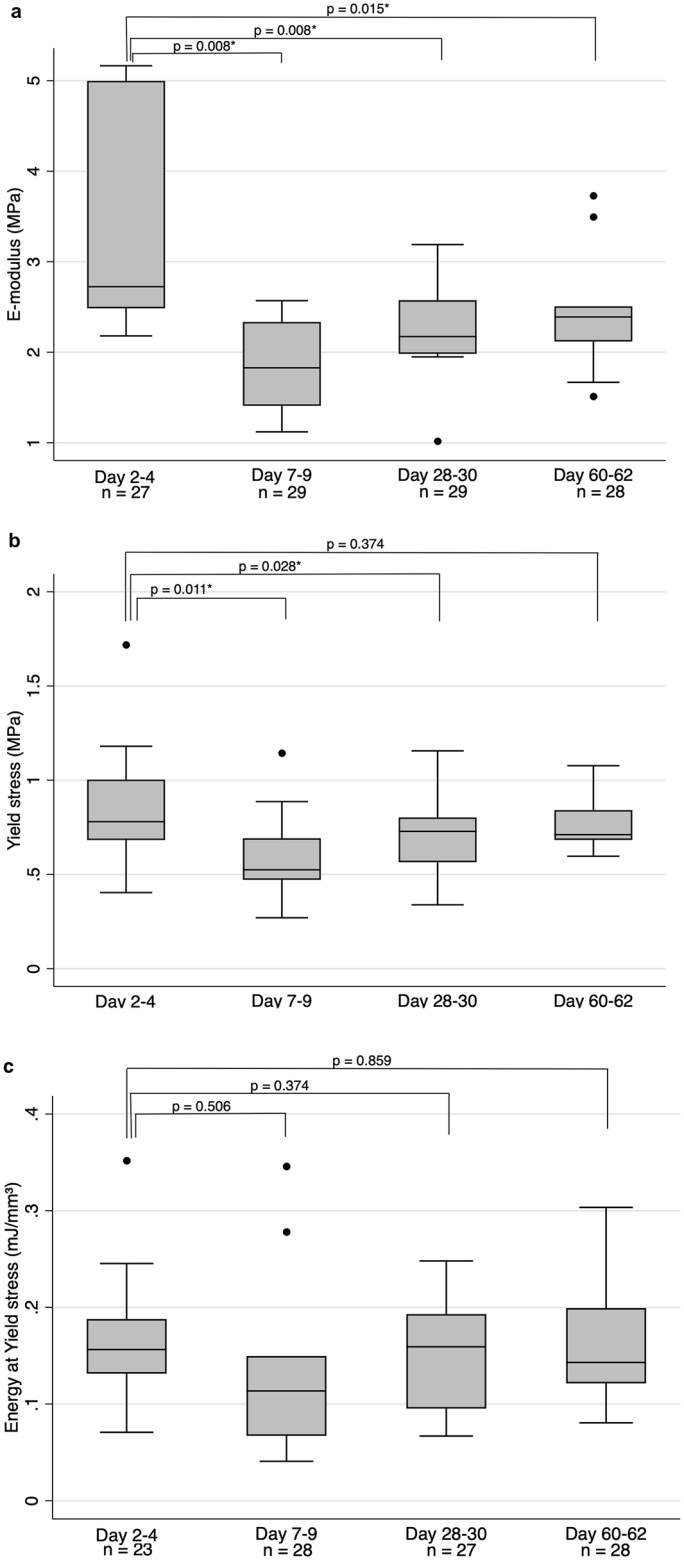
**a** Elastic modulus at different time intervals of decontamination. The middle line is median, the lower and upper axis correspond to the first and third quartiles. The upper whisker extends from the axis to the largest value no further than 1.5 x interquartile range (IQR) from the axis, and the lower whisker extend to the smallest value no further than 1.5 x IQR from the axis. Data beyond whiskers are outlying points that are plotted individually. *p*-values represent results from Wilcoxon Signed Rank test, with individual comparisons between the group of 2–4 days and other groups, **b** Yield stress at different time points. See Fig. 5a for further description, **c **Energy at yield stress at different time points. See Fig. 5a for further description

Elastic modulus, yield stress and energy at yield stress were highest in the reference group of 2–4 days (2.7 (2.5-5.0) MPa, 0.78 (0.68-1.0) MPa and 0.16 (0.13–0.19) mJ/mm^3^ respectively) (Fig. 5; Table 2). The elastic modulus was significantly lower at all later time points, indicating that the stiffness of the tissue has decreased at time intervals of 7–9 days and longer (Table 2; Fig. 5a). The yield stress was significantly lower in the group of 7–9 and 28–30 days compared to 2–4 days, but there was no significant difference in the group of 60–62 days (Table 2; Fig. 5b). There were no significant differences in energy at yield stress between any of the time points (Table 2; Fig. 5c).

**Table 2:** Result from uniaxial tensile testing at different time points

	Day 2–4	Day 7–9		Day 28–30		Day 60–62	
	Median (IQR)	Median (IQR)	*p*-value^a^	Median (IQR)	*p*-value^a^	Median (IQR)	*p*-value^a^
Elastic modulus (MPa)	2.7 (2.5-5.0)	1.8 (1.4–2.3)	**0.008**	2.2 (2.0-2.6)	**0.008**	2.4 (2.1–2.5)	**0.015**
Yield stress (MPa)	0.78 (0.68-1.0)	0.53 (0.47–0.69)	**0.011**	0.73 (0.57–0.80)	**0.028**	0.71 (0.68–0.84)	0.374
Energy at yield stress (mJ/mm^3^)	0.16 (0.13–0.19)	0.11 (0.067–0.15)	0.051	0.16 (0.095–0.19)	0.374	0.14 (0.12–0.20)	0.859

^a^Differences were analyzed using the Wilcoxon Signed Rank test. Groups are compared separately to the group “day 2–4”

Bold values highlight that the result has a *p*-value < 0.05

### Morphology

**Fig. 6:**
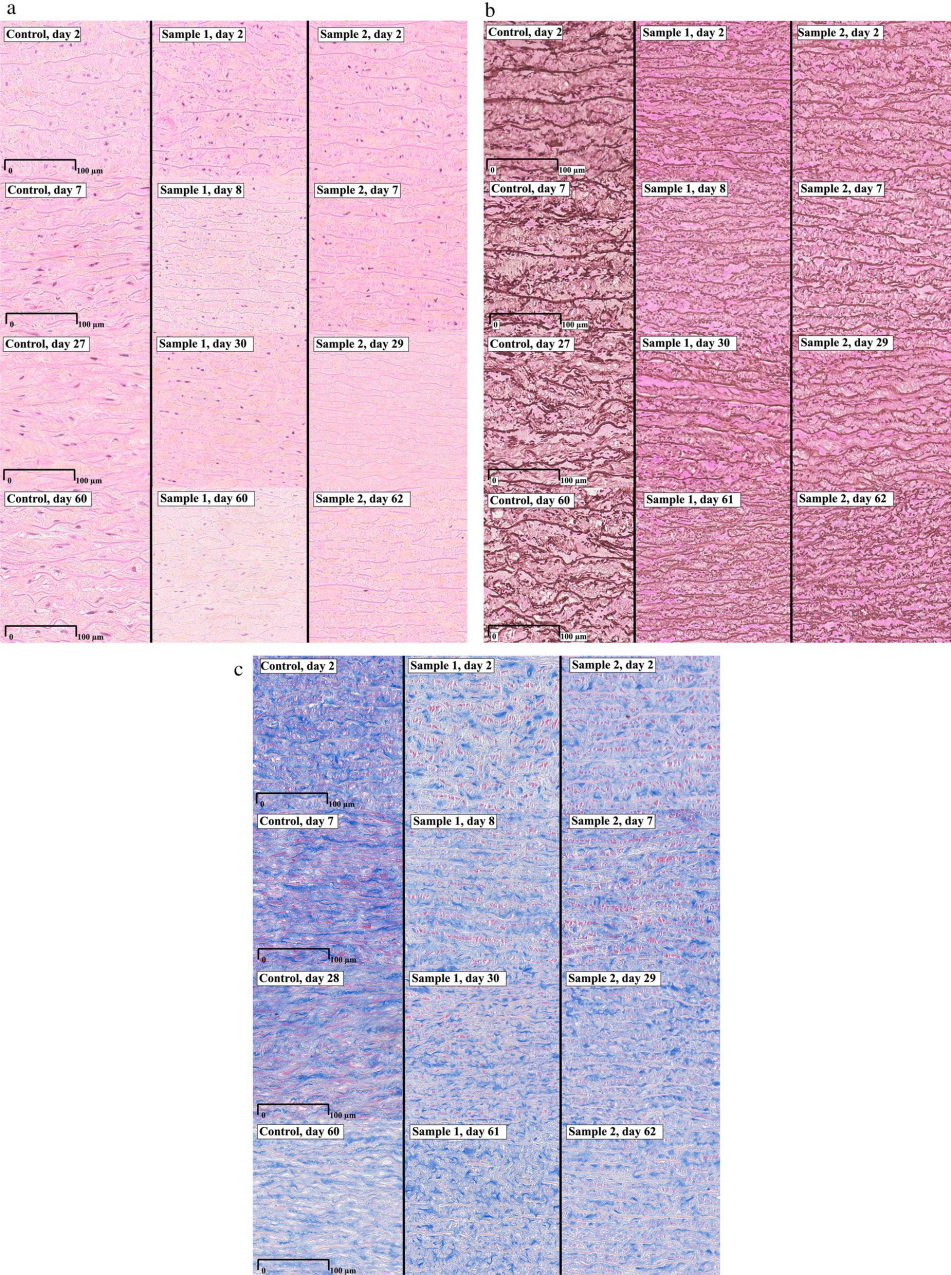
**a** Cellular components at different time points. Stained with Hematoxylin-eosin saffron. Control refers to a sample that has not been cryopreserved or mechanically tested. **b **Elastin components at different time points. Stained with elastica van Gieson. **c** Collagen components at different time points. Stained with Azan

There were no differences in the appearance of cell nuclei in homografts from day 2–4 and 7–9. In one of the homografts that underwent mechanical testing, the tissue was almost completely acellular at day 28 and day 60 (Fig. 6a, sample 2). There were no differences when comparing elastin at different time intervals (Fig. 6b). When comparing collagen, there were no differences between different time intervals. However, the homograft that had undergone cryopreservation and mechanical testing seemed to have collagen fibers with less structure (Fig 6c, Sample 2 and 3).

## Discussion

This study is the first to evaluate the mechanical properties of the homograft in relation to different decontamination intervals. Similar measurements have been made to evaluate other aspects of the homograft procurement process, such as the impact of different ischemic times and different cryopreservation periods (Bester et al. 2018; Fiala et al. 2019). The main results from this study show that elastic modulus and yield stress had decreased at 7–9 days of decontamination compared to 2–4 days of decontamination. Seeing that homograft procurement, preparation, cryopreservation, and thawing were identical to standard protocols, homograft in the group of 2–4 days should be comparable to homografts used in clinical practice.

Tissue banks often aim to keep their procurement process as short as possible but still accepted different maximum time intervals, from three to seven days (total time from donor death until cryopreservation of homografts) (Keitel 2019; Zahra et al. 2019). Extending the time prior to cryopreservation to its extreme seems to affect the homograft in a negative way, seeing that long-term results improved when changing the storage method from a fridge with a maximum of two months storage to cryopreservation in 1987 (O’Brien et al. 1987, 1995). Thus, we do not know how and when the homograft starts to deteriorate in a way that affects its function and quality. This knowledge gap makes it difficult to agree on which time intervals should be accepted. Narrow intervals will ensure homograft quality but lead to difficulties in finding enough homografts to meet current demand. Longer intervals could extend the possible donor pool, providing more time to locate, investigate, transport, and prepare homografts.

Cell viability decreases with a longer preparation time, but this does not seem to affect the durability after implantation (Mochtar et al. 1984; Angell et al. 1989; Niwaya et al. 1995; Gall et al. 1998). In our samples, almost complete acellularity was seen in Sample 2 at day 29 and 62, while the other two samples showed normal cellularity at all time points (Fig. 6a). Studies comparing different ischemic times in relation to long-term performance of homografts have not been able to find any differences between homografts with ischemic times up to 24 h compared to a maximum of 48 h (Meyns et al. 2005; Kalfa et al. 2011; Axelsson and Malm 2018). Recent studies of decellularized homografts have shown promising results, indicating that cell viability in the homograft is not needed to ensure homograft quality at implantation. In contrary, cell-free homografts seem to be more resistant to degenerative processes (Sarikouch et al. 2016; Boethig et al. 2019).

In addition to cells, homograft tissue is composed of elastin and collagen, the major proteins of the extracellular matrix in both vessels and cusps. Elastin is giving elasticity and compliance to the tissue while collagen contributes to strength and stiffness. In a typical stress-strain curve for our samples (Fig. 3), the first part of the slope is less steep (so called ‘toe-region’), followed by a sudden increase in the stiffness at a certain strain-level. This is described by the collagen fibers being crimped at rest, allowing for the tissue to elongate at small forces. When the crimping of the collagen fibers has been fully stretched, the response becomes stiffer (observed by the increased slope). At this point, the collagen fibers are elongated (Burkert et al. 2021). Finally, the collagen network is disrupted, and the tissue fails.

Our results show that both the elastic modulus and yield stress were highest at early time points (2–4 days), and lowest after 7–9 days. A decrease in the mechanical parameters at 7–9 days could indicate a weakening of the collagen fibers, as they provide both stiffness (elastic modulus) and strength (yield stress) to the tissue. Looking at the morphology of our samples, there was no obvious difference between elastin and collagen fibers at different time points (Fig. 6b-c). Cryopreserved collagen fibers (Sample 1 and 2 in Fig. 6c) exhibited less structured fibers at all time points compared to the control sample that was neither cryopreserved nor mechanically tested. Studies have shown that collagen deteriorates after cryopreservation, which could explain this result (Schenke-Layland et al. 2006).

There are no other studies investigating the mechanical properties of the homograft vessel wall after different decontamination intervals. However, there are studies investigating other aspects of the homograft with mechanical testing (Smit et al. 2015; Bester et al. 2018; Fiala et al. 2019). One study that evaluates homograft tissue after different cryopreservation intervals compared to fresh homografts presented similar values of the elastic modulus and tensile strength as in our study (Fiala et al. 2019). Two studies are investigating the mechanical properties of pulmonary homografts from sheep, the first study analyzing homografts at different ischemic times and the second study analyzing homografts with different ischemic times that had been implanted in sheep for 180 days (Smit et al. 2015; Bester et al. 2018). Both studies indicate that there is no difference in mechanical properties of the homograft when prolonging the ischemic time (time before explantation of the heart) to 48 or 72 h. However, studies on human homografts are lacking. These results indicate that homografts keep their structural quality at longer ischemic times, but it is difficult to compare this study to ours since we have investigated a different step of the procurement process.

### Limitations

When performing tensile testing of soft tissues, it is always a challenge to mount the samples well to avoid slipping. We were generally able to avoid slipping, but instead most samples ruptured close to the grips, raising the concern that the grips may have weakened the tissue locally. All samples were mounted in the same way, meaning that this would influence all samples equally. It does generally not affect the measurements of the elastic modulus but could affect the measurement of yield stress and energy at yield stress.

This study included tissue from ten donor homografts, resulting in a small biological group. However, we were able to take 12 samples per homograft, including three replicate measurements at each time point, increasing the sample size to a total of 120 samples, enabling us to reduce variability by paired comparisons.

## Conclusion

This study based on cryopreserved aortic homografts, showed lower values of the elastic modulus and yield stress after 7 days of decontamination at 4 °C. This result could indicate some deterioration of elastin and collagen, resulting in reduced stiffness and resistance to maximal tensile stress. The result suggests that the extracellular components of the homograft could be of further interest when investigating the optimal time intervals for homograft procurement. The clinical significance of these findings remains to be clarified.

## Data Availability

Data is available upon request.

## Abbreviations

EDQM: European directorate for the quality of medicine and health care
NHBD: Non-heart beating donor

## References

[CR1] Angell WW, Oury JH, Lamberti JJ, Koziol J (1989). Durability of the viable aortic allograft. J Thorac Cardiovasc Surg.

[CR2] Axelsson I, Malm T (2018). Long-term outcome of Homograft Implants related to Donor and tissue characteristics. Ann Thorac Surg.

[CR3] Axelsson I, Malm T, Nilsson J (2021). Does microbiological contamination of homografts prior to decontamination affect the outcome after right ventricular outflow tract reconstruction?. Interact Cardiovasc Thorac Surg.

[CR4] Bester D, Botes L, van den Heever JJ (2018). Cadaver donation: structural integrity of pulmonary homografts harvested 48 h post mortem in the juvenile ovine model. Cell Tissue Bank.

[CR5] Boethig D, Horke A, Hazekamp M (2019). A European study on decellularized homografts for pulmonary valve replacement: initial results from the prospective ESPOIR Trial and ESPOIR Registry data. Eur J Cardio-Thor Surg.

[CR6] Burkert J, Kochová P, Tonar Z (2021). The time has come to extend the expiration limit of cryopreserved allograft heart valves. Cell Tissue Bank.

[CR7] Fiala R, Kochová P, Kubíková T (2019). Mechanical and structural properties of human aortic and pulmonary allografts do not deteriorate in the first 10 years of cryopreservation and storage in nitrogen. Cell Tissue Bank.

[CR8] Gall KL, Smith SE, Willmette CA, O’Brien MF (1998). Allograft heart valve viability and valve-processing variables. Ann Thorac Surg.

[CR9] Kalfa D, MacÉ L, Metras D, Kreitmann B (2011). How to choose the best available homograft to reconstruct the right ventricular outflow tract. J Thorac Cardiovasc Surg.

[CR10] Keitel S (2019). Guide to the quality and safety of tissues and cells for human application.

[CR11] Kubíková T, Kochová P, Brázdil J (2017). The composition and biomechanical properties of human cryopreserved aortas, pulmonary trunks, and aortic and pulmonary cusps. Annals of Anatomy - Anatomischer Anzeiger.

[CR12] Lin CH, Kao YC, Lin YH (2016). A fiber-progressive-engagement model to evaluate the composition, microstructure, and nonlinear pseudoelastic behavior of porcine arteries and decellularized derivatives. Acta Biomater.

[CR13] Meyns B, Jashari R, Gewillig M (2005). Factors influencing the survival of cryopreserved homografts. The second homograft performs as well as the first. Eur J Cardio-Thor Surg.

[CR14] Mochtar B, van der Kamp AWM, Roza-De Jongh EJM, Nauta J (1984). Cell survival in canine aortic heart valves stored in nutrient medium. Cardiovasc Res.

[CR15] Niwaya K, Sakaguchi H, Kawachi K, Kitamura S (1995). Effect of warm ischemia and cryopreservation on cell viability of human allograft valves. Ann Thorac Surg.

[CR16] O’Brien MF, Stafford EG, Gardner MAH (1987). A comparison of aortic valve replacement with viable cryopreserved and fresh allograft valves, with a note on chromosomal studies. J Thorac Cardiovasc Surg.

[CR17] O’Brien MF, Gregory Stafford E, Gardner MAH (1995). Allograft aortic valve replacement: long-term follow-up. Ann Thorac Surg.

[CR18] Ross DN, Somerville J (1966). Correction of pulmonary atresia with a homograft aortic valve. Lancet.

[CR19] Sarikouch S, Horke A, Tudorache I (2016). Decellularized fresh homografts for pulmonary valve replacement: a decade of clinical experience. Eur J Cardiothorac Surg.

[CR20] Schenke-Layland K, Madershahian N, Riemann I (2006). Impact of cryopreservation on extracellular matrix structures of heart valve leaflets. Ann Thorac Surg.

[CR21] Smit FE, Bester D, van den Heever JJ (2015). Does prolonged post-mortem cold ischemic harvesting time influence cryopreserved pulmonary homograft tissue integrity?. Cell Tissue Bank.

[CR22] Stradins P, Lacis R, Ozolanta I (2004). Comparison of biomechanical and structural properties between human aortic and pulmonary valve. Eur J Cardio-Thor Surg.

[CR23] Zahra S, Galea G, Jashari R (2019). Significant variation in heart valve banking practice. Eur J Clin Microbiol Infect Dis.

