# Correction to: Impact of storage time prior to cryopreservation on mechanical properties of aortic homografts

**DOI:** 10.1007/s10561-023-10085-1

**Published:** 2023-04-17

**Authors:** Ida Axelsson, Anna Gustafsson, Hanna Isaksson, Johan Nilsson, Torsten Malm

**Affiliations:** 1 Tissue Bank Lund, Baravägen 37, 22242 Lund, Sweden; 2 Department of Cardiothoracic Surgery, Skane University Hospital, Lund, Sweden; 3 Department of Clinical Science, Cardiothoracic Surgery, Lund University, Lund, Sweden; 4 Department of Biomedical Engineering, Lund University, Lund, Sweden; 5 Department of Translational Medicine, Artificial Intelligence and Bioinformatics in Cardiothoracic Sciences, Lund University, Lund, Sweden; 6 Pediatric Cardiac Surgery Unit, Children’s Hospital, Skane University Hospital, Lund, Sweden

**Correction to: Cell Tissue Bank** 10.1007/s10561-023-10079-z

In the original publication of the article, the Fig. 5c was missed.

**Fig. 5:**
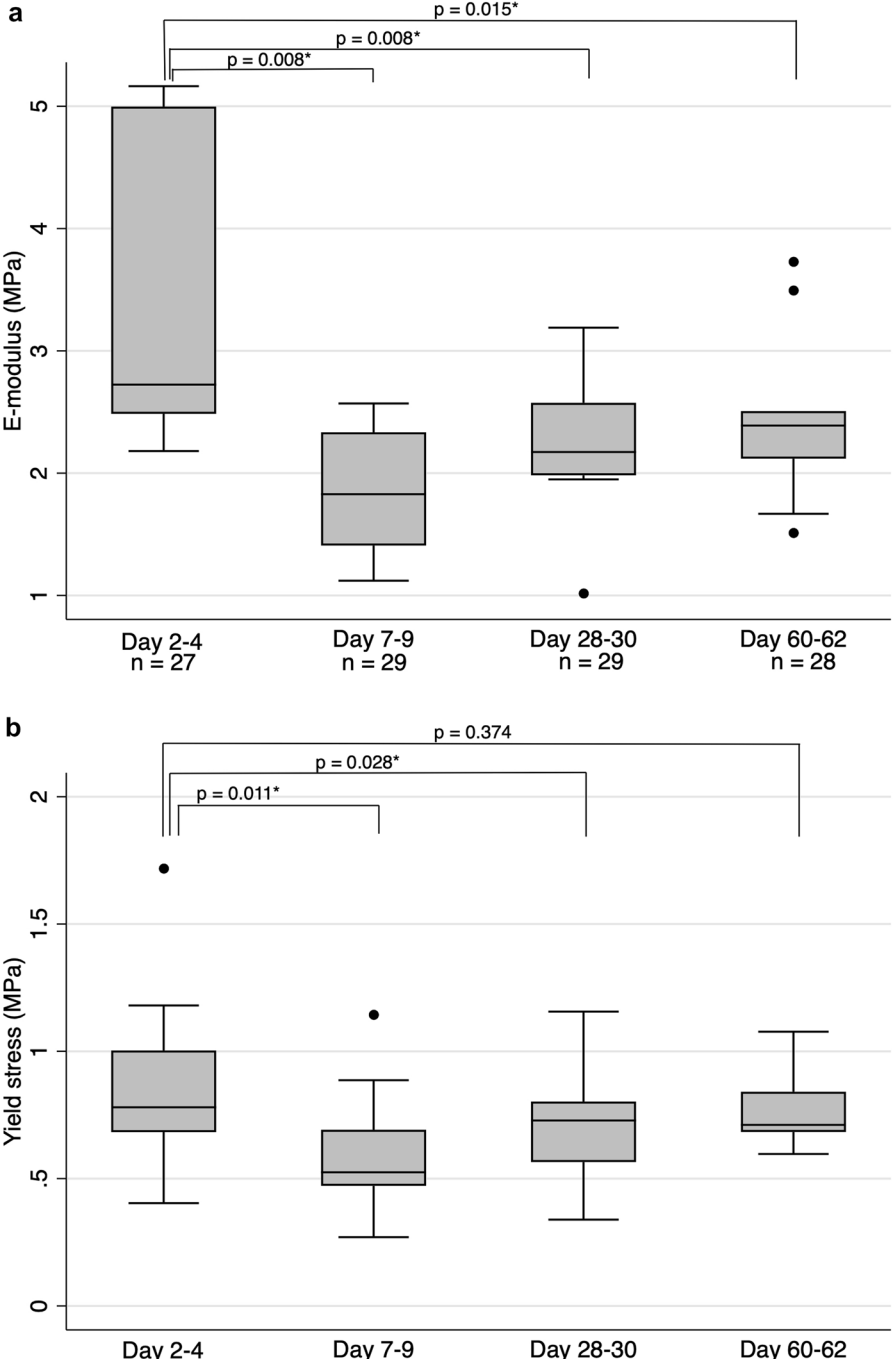

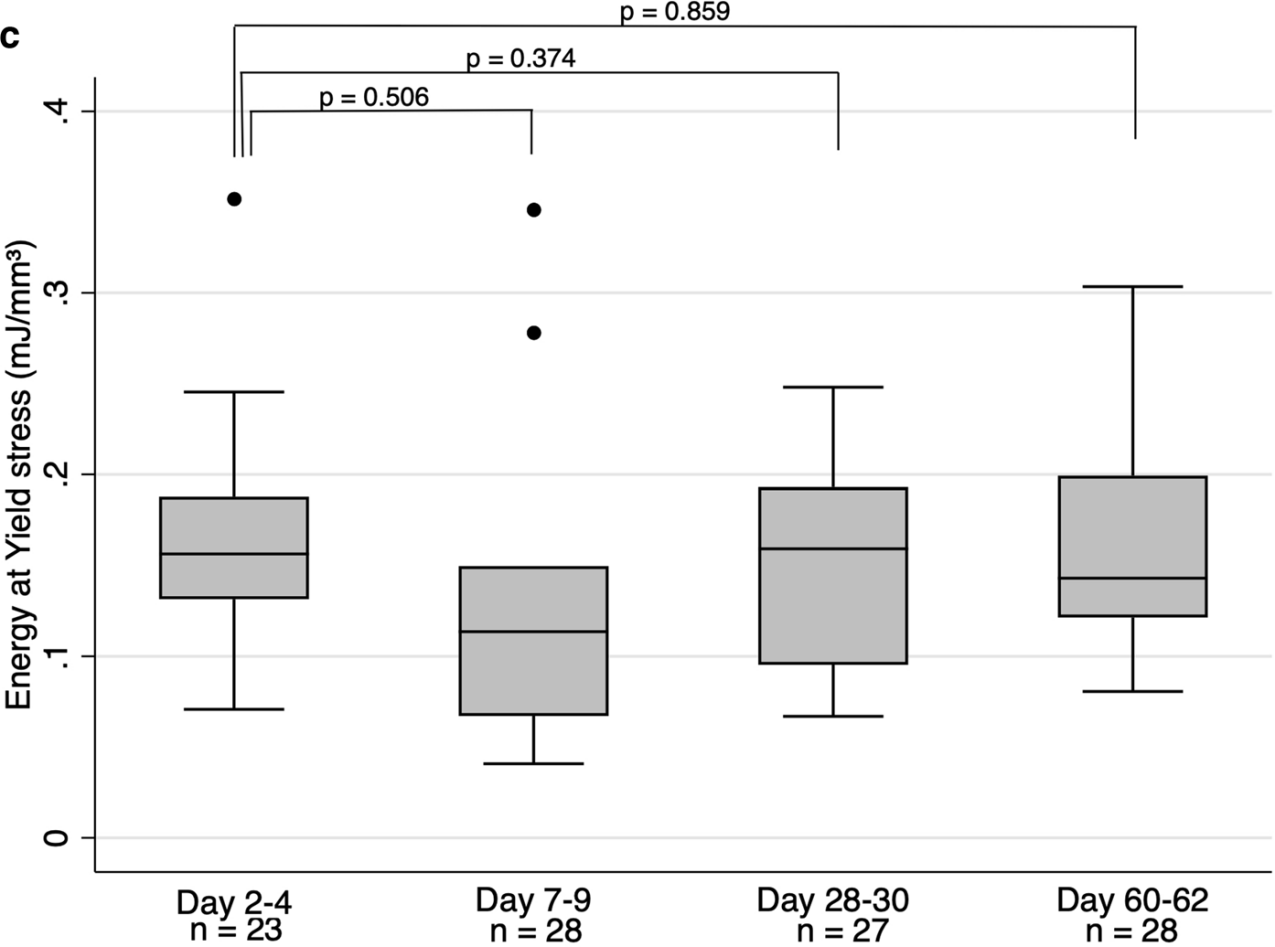
**a** Elastic modulus at different time intervals of decontamination. The middle line is median, the lower and upper axis correspond to the first and third quartiles. The upper whisker extends from the axis to the largest value no further than 1.5 x interquartile range (IQR) from the axis, and the lower whisker extend to the smallest value no further than 1.5 x IQR from the axis. Data beyond whiskers are outlying points that are plotted individually. *p*-values represent results from Wilcoxon Signed Rank test, with individual comparisons between the group of 2–4 days and other groups, **b** Yield stress at different time points. See Fig. 5a for further description, **c **Energy at yield stress at different time points. See Fig. 5a for further description

The complete Fig. 5 is given below.

The original article has been corrected.

